# Supplementary figures and images for: Both trust in, and polarization of trust in, relevant sciences have increased through the COVID-19 pandemic
Source: PLoS One. 2023 Mar 23;18(3):e0278169. doi: 10.1371/journal.pone.0278169 (PMC10035814; doi:10.1371/journal.pone.0278169)

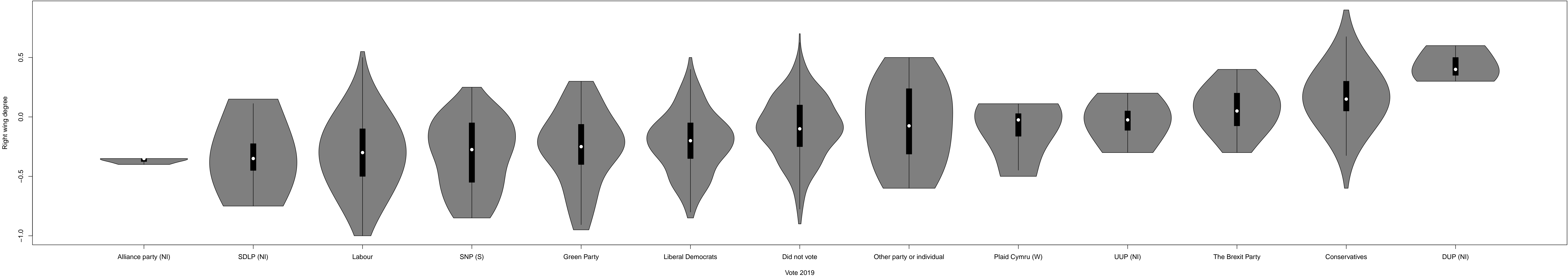

Supplement: S1 Fig — (PDF) [file pone.0278169.s002.pdf]
