# Supplementary material for: Both trust in, and polarization of trust in, relevant sciences have increased through the COVID-19 pandemic
Source: PLoS One. 2023 Mar 23;18(3):e0278169. doi: 10.1371/journal.pone.0278169 (PMC10035814; doi:10.1371/journal.pone.0278169)
Supplement: Attachment — Submitted filename: Responses.docx [file pone.0278169.s003.docx]

Reviewer #1:

Abstract: Needs to be curtailed and specific.

**Response: We have shortened and made the questions more explicit. We hope this addresses this concern**

Introduction and discussion can be strengthened by including the following points:
1. include a sentence on origin, transmission of COVID-19 (refer and cite: doi: 10.1136/postgradmedj-2020-138234)
2. The role of government in combatting COVID-19 in comparison with the other regions (refer and cite: doi: 10.3389/fpubh.2022.844333).
3. The role of social environmental predictors of COVID-19 in general (refer and cite: doi: 10.3390/vaccines10101749)
4. The effect of vaccine hesitancy in COVID-19 and its effects in trust in science (refer and cite: doi: 10.3390/vaccines9101064)
5. The calibre of predictors in COVID-19 (refer and cite: doi: 10.1136/postgradmedj-2021-141365)

**Response: we now include all these references in either introduction or discussion**

Materials and methods: Explain more in detail about the survey, the target population, inclusion and exclusion criteria, if any missed cases were included?

**Response: we now considerable add to the Method statements regarding the survey. The target population was previously defined (but hidden perhaps). We now state that all responses passing QC were analysed.**

Statistics: divide the covariates into dependent and independent variables.

**Response: an explicit statement to this effect is now included**

Mention if bias was generated, and if yes, how was it limited/regressed.

**Response: we add much detail on the metrics of the match between the intended population and the actual sample. The sample is in fact unusually representative. We detail some of the reasons for this in the main text but further details are available in S Materials 1.**

The importance of choosing the statistical relevance should be addressed.

**Response: We now make a statement that we chose to perform non-parametric statistics as we determined that some distributions were deviant from normal.**

Limitations and strengths need to be included in the end of discussion.

**Response: we now have a full subsection on caveats and limitations at the end of the discussion (we had only limited mention before)**

Reviewer #2:

The manuscript should include more figures and illustrations.

**Response: the manuscript has 5 figures, one to illustrate each of the key results. We are not sure what else the referee might be requesting**

To add more details about survey analysis.

**Response: we agree and now add substantially more detail to the methods section in this regard**

Was the survey validated? was a pilot study done?if yes those details need to be included.

**Response: we now state that the questions were taken or lightly adapted from prior benchmarked questions (e.g. from Wellcome surveys, prior Kantar surveys). As such, a pilot was considered unnecessary. We now state as much. Thank you for bringing this to our attention.**

The regional comparison should be included.

**Response: we aren’t certain what the referee is considering here. We now however discuss how our results (e.g. as regards vaccine attitude) compare with comparable sampling in Germany and India. We note that for the most part covariates are not replicated between regions. If the referee is asking us to consider intra UK variation, then we are hesitant as a) the sample size isn’t large enough and b) we are extremely reluctant to provide further statistics if we do not have a good motivation to perform such statistics (such is the nature of P hacking). We hope the new discussion of regional differences addresses the referee’s concerns.**
